# Who needs what from a national health research system: lessons from reforms to the English Department of Health's R&D system

**DOI:** 10.1186/1478-4505-8-11

**Published:** 2010-05-13

**Authors:** Stephen Hanney, Shyama Kuruvilla, Bryony Soper, Nicholas Mays

**Affiliations:** 1 Health Economics Research Group (HERG), Brunel University, Uxbridge, UK; 2 Department of International Health, Boston University School of Public Health, USA; 3 Health Services Research Unit, Department of Public Health and Policy, London School of Hygiene and Tropical Medicine, London, UK

## Abstract

Health research systems consist of diverse groups who have some role in health research, but the boundaries around such a system are not clear-cut. To explore what various stakeholders need we reviewed the literature including that on the history of English health R&D reforms, and we also applied some relevant conceptual frameworks.

We first describe the needs and capabilities of the main groups of stakeholders in health research systems, and explain key features of policymaking systems within which these stakeholders operate in the UK. The five groups are policymakers (and health care managers), health professionals, patients and the general public, industry, and researchers. As individuals and as organisations they have a range of needs from the health research system, but should also develop specific capabilities in order to contribute effectively to the system and benefit from it.

Second, we discuss key phases of reform in the development of the English health research system over four decades - especially that of the English Department of Health's R&D system - and identify how far legitimate demands of key stakeholder interests were addressed.

Third, in drawing lessons we highlight points emerging from contemporary reports, but also attempt to identify issues through application of relevant conceptual frameworks. The main lessons are: the importance of comprehensively addressing the diverse needs of various interacting institutions and stakeholders; the desirability of developing facilitating mechanisms at interfaces between the health research system and its various stakeholders; and the importance of additional money in being able to expand the scope of the health research system whilst maintaining support for basic science.

We conclude that the latest health R&D strategy in England builds on recent progress and tackles acknowledged weaknesses. The strategy goes a considerable way to identifying and more effectively meeting the needs of key groups such as medical academics, patients and industry, and has been remarkably successful in increasing the funding for health research. There are still areas that might benefit from further recognition and resourcing, but the lessons identified, and progress made by the reforms are relevant for the design and coordination of national health research systems beyond England.

## Introduction

While there is a long history of scientists and physicians conducting health or medical research, a health research system is a newer concept. A national health research system has been defined as 'the people, institutions, and activities whose primary purpose is to generate high quality knowledge that can be used to promote, restore, and or maintain the health status of populations. It can include the mechanisms adopted to encourage the utilization of research' [1]. This definition encompasses a wide range of actors and approaches from the public and private sectors, academia, charitable foundations, and civil society who all have some stake and interest in health research and its utilisation.

In practice, it is difficult to draw boundaries around such research systems and the task is made more complicated by changes in the ways in which knowledge is generated by scientific research. The classic and 'internalist' model of scientific research - termed Mode 1 - posits that science has its own structures and processes, and therefore should determine its own priorities [2,3]. This concept has long been debated, and is increasingly challenged by the growing acceptance of the argument that science, state, and society do, and should, interact with, and influence, each other [4-7]. A complex range of stakeholders influence how research is defined, conducted and used. The term Mode 2 research is used to reflect research that is conducted specifically with a view to being applied on behalf of society, the state and the economy [3], and, in this sense, encompasses both research and development (R&D). Because we are taking this broad perspective, we use the terms 'research' and 'R&D' interchangeably in this paper. Others also argue that science is practised in a diffuse and unbounded social context - an *'agora' *[7] - that is an open market place or network, 'with many different actors, political and economic interests and competing scientific knowledge claims' [8]. Reflecting this complexity in the nature of research, there is often also a range of health research systems within any country that seek to achieve specific objectives. Considering *national health research systems*, there is general agreement that one of the main objectives should be to ensure that these diverse actors and health research systems collaborate and that the system is managed in a way that promotes both good science and the public good [1,9].

Following this Introduction, the paper has three main sections:

1. First, we describe the needs that key stakeholders in any health research system are likely to have.

2. Second, we focus on how the research system funded by the English Department of Health over the last four decades attempted to meet the needs of various stakeholders.

3. Third, we analyse, from both stakeholder and historical perspectives, how a more coherent national health research system is gradually emerging in England, and we attempt to draw some lessons about best way to build a health research system to meet the needs of diverse stakeholders.

To conduct this analysis, we reviewed the overlapping literature on stakeholder needs and that on the history of health research reforms in England. The long history of developments related to publicly funded health research in England is comprehensively addressed by Shergold and Grant [10]. Our paper draws on this and other detailed accounts of specific phases and developments of the R&D system of the English Department of Health (DH) [11-15]. Key phases of reform in this system have concentrated on the needs of different stakeholders to varying degrees. In particular, we draw extensively on the seminal analysis by Maurice Kogan and Mary Henkel of the reforms in the 1970s to the research system of the English DH, the full title of which at that time was the Department of Health and Social Security. First published in 1983, this analysis emphasised the 'multi-modal' nature of stakeholders' roles and interactions, and the importance of 'interface mechanisms' between stakeholders and the health research system [11]. This line of analysis was further developed by Hanney et al, in the context of a World Health Organization coordinated international exercise to analyse national health research systems [16]. The 'Interfaces and receptor model' that was developed highlights health research system interfaces and the related tasks of research specification, commissioning, conduct, synthesis and appraisal, dissemination, application, and feedback. In the English system this range of tasks was explicitly recognised in the reforms of the early 1990s, which covered both research and development [17]. In drawing lessons from each phase of reform, we highlight points that emerged from contemporary reports and also use the above frameworks to identify and analyse issues.

While this analysis is situated in the English health R&D system, and specifically in the part of the system covered by the government's health department and the health care system, the issues discussed are relevant for the design and coordination of national health research systems far more widely, and might have some applicability to large systems within national systems, for example the US Department of Veterans Affairs. Therefore, we provide generic definitions of specifically English organisational arrangements, and also aim to draw lessons from the reforms to the English health R&D system that could also have more general applicability elsewhere. This is especially so because there is a general trend in many countries towards the involvement of more stakeholders in research systems, and hence the need for greater coordination across any such system [18,19].

## Stakeholders in Health Research Systems

At the core of any health research system are the researchers who undertake the research, the diverse bodies that fund it, and those organisations that host it. Then there are a range of other interests that relate to health research in various ways. In a stakeholder analysis it is useful to focus on the key groups who are seen as the recipients of the research and identify what they are likely to need from the health research system. There have been successive reforms of that part of the national health R&D system that is funded by the English DH over the last four decades. To varying degrees, each of these successive phases has given particular attention to the needs of specific stakeholder groups:

• policymakers (and managers),

• professionals in the health-care system,

• patients and the wider public, and

• industry.

**Figure 1:**
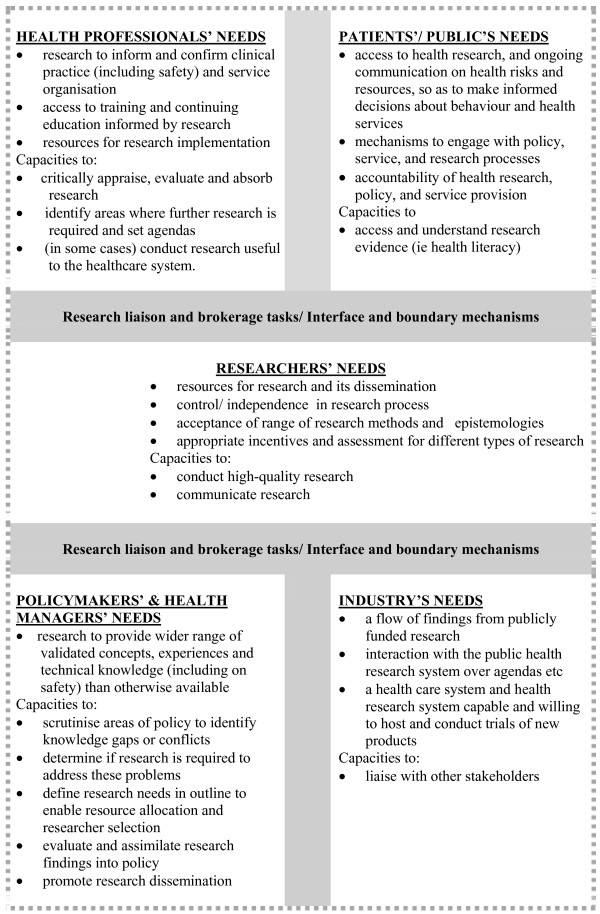
**Examples of needs in relation to publicly funded health R&D**.

The stakeholders are shown at the four corners of Figure 1 which summarises their needs. All the groups interact with and influence each other, but consistently the fifth stakeholder group, the researchers, who are at the centre of Figure 1, feel the pressure from many of the other stakeholders. The needs of these five key stakeholder groups are described in detail below, but first we describe the context in which they operate within the English system.

### Context in which key stakeholders operate within the English system

In recent years the Treasury (which is the UK equivalent to departments or ministries of finance in other countries) has become increasingly interested in performance and outputs when making the overall decisions about levels of public expenditure and allocations to the various government departments (the term by which ministries are known in the UK). Such departments include several that receive money to fund health research. Whilst the health department has remained a constant player over many years, and funds research directly through its own R&D Division and through the National Health Service (NHS), the other departments involved in funding health research have varied. Most universities in the UK receive considerable public funding but they are charter institutions. This means they are not strictly public sector organisations: 'their status has been that of almost wholly independent institutions but deferring to public policies which largely constitute the conditions under which the bulk of their resources have been secured' [20]. The universities are funded for both teaching and research through funding councils that at various times during the last four decades have reported to either the main education department, or to a separate department, or to a section of a department dealing with business. In addition, the research councils in England provide a separate but major source of public funding for research: in the health field the Medical Research Council (MRC) was created in the early 20th Century [10]. Like the universities, the research councils have traditionally enjoyed a considerable degree of autonomy [20]. The government department to which the research councils report has changed frequently and has included: Education; Industry; Business; and the Cabinet Office, which is the central co-ordinating department in British government.

The role of the private and charitable sectors in health research is increasingly and explicitly recognised. In terms of expenditure it was estimated in 2005 that the annual private sector, ie industry, medical research funding was about £5,000 million in the UK, compared with £1,700 million from public sources and £650 million from the medical research charities [21].

In terms of the locations where the research is conducted, there is recognition in recent reforms that health researchers work at multiple organisational levels in the publicly-funded health R&D system, each with different stakeholder considerations, including in universities and in hospitals (and often in the medical schools that combine the two), and in units funded by the main research council in this field, the MRC. Furthermore, there is an extremely large sector of health R&D conducted in the laboratories of industrial companies. Most of the charitable and some of industry's funding for science goes to research conducted in public sector institutions.

There is considerable interest in science policy in the legislature. However, there is a long history in the UK, as expressed in the Haldane Report [22], of the government delegating autonomy to the research councils over which projects they should fund, and also of legislation governing research being relatively limited. Of the two chambers in the UK Parliament it is possibly the unelected and generally less powerful chamber, the House of Lords, that has had the most influence over the way science policy develops. This is partly because of the role of its Science and Technology Select Committee, which consists of some of the most senior scientists in the country, often appointed to the House of Lords because of their scientific achievements [12].

There is increasing public interest in medical research and many people obtain science and health information mainly from the mass media [23,24]. There is also growing evidence that the mass media can influence health policy agendas, the utilisation of health research, and health behaviours [25]. It is increasingly seen as desirable for the health R&D system to devote some resources to attempting to enhance the health literacy of both the general public and parts of the mass media.

There are further complications. Some individuals play more than one role, and are therefore members of more than one stakeholder group. For example, many medical academics are both practitioners and researchers. The various stakeholders relate to each other, and with health R&D, in various settings, including ones related to governmental, managerial and clinical policy making. Furthermore, each group can also be 'multi-modal', meaning that there can be great variations between, for example, policymakers from different parts of the Department of Health (DH) or between the skills and attitudes of scientists working in different branches of health research [12]. Whilst health professionals can be referred to as one stakeholder group, in practice, there can be great differences between those filling the various roles, and such differences can be reflected in how they relate to research [26].

Deciding where to draw the boundary for an analysis of any part of the health research system is never clear-cut, and where possible the analysis should consider the governance structures for the funding and management of health research, and discuss some of the challenges faced. In this analysis we primarily focus on the health R&D conducted in public organisations (counting universities as 'public organisations') and funded through the English Department of Health and the health care system it provides (ie the NHS). But we also consider the wider range of stakeholders that influence, and draw on, that research system. Our analysis, however, goes further. We describe the developments in the English DH's system of research funding since the 1970s that have now led to the creation of a national health R&D system that seeks both increasing coordination across the whole publicly funded system, and improved liaison with industry and with the medical research charities.

The five key stakeholder groups on which this analysis focuses are presented below.

### Policymakers and managers in the health care system

For the purposes of our analysis, NHS managers are classified alongside policymakers, as depicted at the left-hand lower corner of Figure 1. This is based on the interlinked nature of their needs from health research systems. The underlying assumption is that policies and managerial practices which are research-informed will be better than otherwise would have been the case [12,16]; research exposes policymaking to a wider range of validated concepts and experiences than those that might otherwise be available in time-limited and politically constrained processes of policy deliberation. Research legitimises some policies and throws legitimate doubts on others.

Different types of research are likely to be required to inform different types of policies in health care. These include, for example, 'legislative, administrative, or clinical' policies [27]. The utilisation of research in policymaking can be conceptualised in a range of ways [28] and a three-fold categorisation is sometimes adopted: instrumental, conceptual or symbolic [29,30]. Instrumental use involves research findings being directly used in policy formulation. Conceptual use refers to a type of enlightenment mode of utilisation in which the ideas from research gradually seep into the way in which issues are thought about. Finally, symbolic use of research occurs when it is used to support a position already taken, which may be to continue with existing policies.

In terms of clinical policies, the technical issues around the effectiveness and cost-effectiveness of clinical interventions are key considerations. It is often thought that research has more impact, at least in a more readily identifiable instrumental way, on clinical policies than it does on legislation - although there is now increasing evidence of instrumental impact with regard to the latter [31]. It also is likely that there is longer-term conceptual impact on the way in which legislative and administrative policies are viewed, but it can be difficult to link this to specific research.

The experience of Kogan and Henkel, and the message of many subsequent analyses, is that policymakers need to develop various capabilities in order to gain the benefits from research [12,32,33]. These include capabilities to: scrutinise areas of policy to identify knowledge gaps or conflicts; determine if research is required to address these problems; and define research needs in outline to enable resource allocation and researcher selection. These activities often take place at the project specification and commissioning interface between research and policy where it is claimed that a collaborative approach between policymakers and researchers is likely to productive [4].

Long-term relationships developed between researchers and potential users in the health care system are seen as a productive way of developing collaborative approaches, but as Kogan and Henkel showed there can be many obstacles to doing this consistently [11], including the frequent turn-over of staff in policymaking roles. Increasingly researchers and policymakers in the UK, as elsewhere, are developing networks that contribute to research agenda development [31].

Just like policymakers, health service managers and clinicians, including those who make decisions at local levels, are likely to benefit if they have the capacity to help identify what they need from research through scrutinising areas of service organization or provision and defining suitable topics. For those working at local levels, however, there have traditionally been fewer opportunities to be involved in the commissioning process for research to meet the needs of their specific organisations or services, although this is now beginning to change.

Once the research has been produced, policymaking and managerial systems also need the capabilities to evaluate these findings and assimilate them into national and local policy. Various mechanisms and capabilities can help to promote research dissemination and implementation [12,16]. These include: developing appropriate brokerage roles to assist the transmission of the findings from the researchers to the relevant policymakers; ensuring that the bodies that receive and use the research findings, (i.e. the 'receptor bodies') have the requisite organisational capacity to absorb the findings; and the provision of relevant training for policymakers, administrators and managers [12,16]. How far all these capabilities are the responsibility of the health research system to provide is debatable, but one area in which health research systems have increasingly been attempting to meet the needs of policymakers and managers is through the development of capacity to undertake systematic reviews, and interpret the results from them. A recent international review of health research funding agencies' support and promotion of Knowledge Translation found, however, considerable variation in their level of engagement in such processes [34].

In some health care systems there have recently been more assertive attempts to generate policies to influence the behaviour of health care professionals when conducting their clinical activities. For example, in England, the Department of Health established a series of ad hoc committees to draw up National Service Frameworks that included recommendations for the organisation of health care in a specific field, such as diabetes, or for a specific group, such as older people. The DH also created the National Screening Committee (NSC) that generates policies about what types of screening will be provided within the NHS, and the National Institute for Health and Clinical Excellence (NICE) that issues policies in the form of guidance about whether specific treatments should be funded. Clinicians and commissioning bodies in the Health Service are increasingly obliged to follow such guidance. NICE also issues guidelines which provide recommendations for clinical behaviour in broader areas of health care. Some of the organisations that generate such policies, including NICE in England, can be described as receptor bodies for research [12,16]. International evidence suggests that Health Technology Assessments will not necessarily be much used unless a policymaking, or receptor, body is properly established to use the research [16].

There is a complex interplay between developing the capacity of people working in existing policymaking, or receptor, bodies to receive and use research findings, and going further and specifically creating new receptor structures to undertake evidence-based policymaking. In terms of providing training to develop capacities, the Canadian Health Services Research Foundation runs the Executive Training for Research Application programme designed to, 'improve the receptor capacity of both health service executives and the organizations in which they work' [35].

### Professionals in the health care system

Professionals working in the health care system need research to inform and confirm clinical practice, including research on the safety, effectiveness and cost-effectiveness of therapies. Similarly, professionals who also have responsibility for service organisation benefit from research findings to inform their decisions. Health care systems and organisations are immensely complex and there are many ways in which professionals, at an individual level or as part of clinical teams, can access, or be exposed to, research findings. These include through journals, online resources, conferences and through receiving training and continuing education that is informed by research. There has been extensive international debate about how professionals should be encouraged to access to research findings, and be informed by them [36].

There is an increasing realisation that not only do individuals need good access to research but also that work is needed at the level of the health care organisation and the system to encourage the implementation of health research [37,38]. In addition to the behaviour of individuals and teams, and the cultural changes that can occur at organisational and structural levels, there are also, as noted above, increasing attempts within some health care systems to influence clinician behaviour through policies made at a national level, which include clinical guidelines produced by professional bodies. Professionals who want to implement research findings also need the resources to do so, and capacities such as the ability to critically appraise, evaluate and absorb research. Training professionals to acquire these capacities has, at times, been seen as the responsibility of the health research system, for example, by running critical appraisal courses [39].

Turning to the production of research, there are several roles professionals can play. First, it is likely that professionals will be most responsive to research if they believe it is relevant to their needs. This is more likely to be the case if they have been able to exploit opportunities to help identify areas where further research is required, and thus to help set agendas. Second, and more obviously, the medical academics and other health professionals who engage in research need capacities and opportunities to conduct such research, as is discussed below in the sub-section on the needs of researchers.

One of the benefits of funding research widely in a health care system, so that many of the professionals have some experience of being involved in research, is that it is thought to increase the capacity of people working in that system to take on board and utilise research findings in general. This concept of increasing the capacity of the health care system to absorb research (called the absorptive capacity) through conducting research has long been built into ways of assessing the impact of health research [40].

### Patients and the general public

Increasingly patients and the general public wish to have access to research evidence so as to make informed decisions about their own care and behaviour in relation to health services, and to have greater understanding of health risks [5,41]. Indeed, the constitution of the World Health Organization states: 'An informed opinion and active cooperation on the part of the public are of the utmost importance in the improvement of the health of the people.' [42].

But to be able to gain these benefits, patients and the public need to have various capacities, including health (and perhaps health research) literacy, so as to be able to access and understand research evidence [43]. As noted, a systematic review found that the media are leading sources of health information targeted by those aiming to influence behaviour of the public (as well as of health professionals) [25]. This increases the importance of health research systems exploring the best ways to engage the mass media, and to consider the most appropriate forms of health research communication. This is important not only to ensure the widest possible coverage for evidence-based health research findings, but also to counter the dangers of misinformation, for example from inadequate research.

Patients and members of the public, and their representatives, either wish, or at least are increasingly being encouraged, to engage with research processes such as agenda setting and commissioning [44,45]. These moves partly reflect broader socio-political changes, in this case for more participatory forms of decision-making [5], but, especially when it was first being undertaken, there was often confusion about what patient involvement entailed [46]. A systematic review on consumer involvement in health research agenda-setting published in 2004 claimed ongoing collaboration had the greater impact compared with one-off consultation, but concluded that, 'what we know about the advantages and disadvantages of methods of involving consumers in agenda setting rests on weak short-term evidence and almost entirely speculative long-term evidence' [47]. Furthermore, even by 2009 the latest review refers to gaps in the evidence about the impact of public involvement on research funding and commissioning [45].

To be able to participate fully patients need to develop appropriate capacities, and several health research systems have explored ways to provide training [48] and develop innovative techniques [49]. Furthermore, attempts to increase the involvement of members of the public also require flexibility in the approach adopted by the members of research commissioning panels from traditional scientific backgrounds. There is an important need for trust and respect on both sides, and it is recommended that new protocols are adopted in such circumstances [50].

As with all groups of stakeholders, patients do not act as a homogenous group. For example, organised patient groups tend to push for more research in their particular field, and the lack of a strong advocacy group for public health research may have contributed to the traditionally low levels of funding in that area.

### Industry

The pharmaceutical and medical device industries also need a flow of findings from publicly funded research, in terms of new knowledge, research material and techniques including assays, epidemiological data, etc. In the commercial world in general, a considerable proportion of the investment made by industry in its own laboratories is to ensure sufficient absorptive capacity; i.e. the ability to assimilate and exploit external knowledge [51]. In order to ensure this exchange of knowledge occurs more systematically, industry works with researchers to develop mechanisms such as Material Transfer Agreements which govern the transfer of tangible research materials between two organisations, such as industry and a part of the health research system, when the recipient intends to use it for his or her own research purposes.

In order to maximise the benefits from publicly funded research, industry is increasingly active in seeking to influence the agenda of the publicly-funded health research system at an organisational level, and to develop links with individual researchers and research groups. Industry is also interested in mechanisms whereby they can interact with the other stakeholders of health research systems.

Furthermore, industry needs a health care system and health research system capable and willing to host and conduct trials of new products, and here high income countries are facing an increasing challenge from low and middle income countries. Most countries have some type of drug regulatory agency that decides which products to approve for use in their country, or for use in their publicly-funded health care system. Industry often takes considerable steps to influence the health research system mechanisms put in place to provide data for the drug regulatory agency.

There are inevitably concerns among the other stakeholders about the influence of the private sector given that roles and interests of the public and private sectors are not always congruent or clear. For example, a systematic review found that trials funded by the pharmaceutical industry tended to have outcomes that favoured products made by the sponsoring companies [52]. Furthermore, there are particular concerns about the part played by the tobacco industry in trying to undermine independent research on the health dangers of smoking [53].

### Researchers

It is in everyone's interests that the central stakeholders, i.e. the researchers themselves, are adequately supported by others in any health research system. Nevertheless, there have been occasions when the money allocated for research within health care systems has been vulnerable to being siphoned off to be used by other parts of a hard-pressed health care system. For researchers, a key consideration during periods of reform to the health research system is what will happen to the level of resources available for their research.

Researchers also need broad acceptance for the range of research methods they might use and the accompanying epistemologies. Here there can be problems, particularly as the scope of health research has widened and attempts are made to introduce a wider range of qualitative methods [54].

All researchers also need appropriate incentives. A more complex issue is that researchers need to feel that the assessment of their research reflects the basis on which it was commissioned. In particular, health services researchers have long felt that the criteria used to assess policy-relevant research have not always been appropriate [12,31]. Amongst analysts of research systems there is a wider recognition that if researchers are now operating in an *agora *- an open market place of ideas - then the criteria on which they are assessed should reflect this [8]. However, in practice, the needs of applied researchers have often not been taken into account when academic assessment systems were devised, either by government or by the universities themselves.

Linked to the above points, but often potentially in conflict with the needs of other stakeholders, is the need that most researchers feel for a reasonable level of control over the methods used - at least during the research process itself. It has traditionally been claimed, especially in the Mode 1 model of research (i.e. the classic or internalist model often found in basic research), that the most good will come to society when researchers are left free to determine their own research agendas and priorities, driven by the imperatives that come from the unfinished business in the science [2]. But, as noted in the introduction, this is now a matter of contention. In contrast to those who fear losing control of the research agenda, some applied researchers increasingly claim that a collaborative approach is likely to be more productive than researchers either devising their own agendas or simply being invited to do specific research to meet the needs of policy 'customers' [4]. They claim that a collaborative approach will result in research for which there will be potential users [4], but the approach requires appropriate structures to be created. Within this, there must be scope for the researchers to influence the agenda, particularly in relation to defining researchable topics, and have considerable autonomy over the methods to use. There is mounting evidence that some programmes are able to adopt a successful collaborative approach [55].

Researchers need various capabilities. These include the capacity to conduct high-quality research and, it is increasingly argued, to communicate research findings and collaborate with policymakers. For these capabilities, more so than those of any other stakeholders, the health research system has a responsibility to meet the need.

## Key Phases of English health R&D Reform

This next section of the analysis provides an overview of the four main attempts to reform aspects of the English health research system, and discusses how these reforms addressed the stakeholder needs identified in the preceding section. Whilst the needs of all stakeholders featured to some degree throughout, each phase in the Department of Health's R&D reforms in England over the last four decades can be viewed, and were presented at the time, as emphasising the needs of different stakeholders:

1. *'Government as consumer of research' *- The Rothschild reforms in the 1970s focused on strengthening links between government and science, and developed clear 'customer-contractor' relationships between policy officials and researchers for applied research [56].

2. *'The National Health Service (NHS) as consumer of research*' - The NHS R&D/Peckham reforms from the early 1990s were directed towards integrating research and development into the NHS through trying to improve the links with its policymakers, managers and professionals - to improve health and health care, and led to the establishment of the NHS R&D programme [57].

3. *'Multiple stakeholders including patients as consumers of research' *- The health department's R&D strategy proposed in 2005, *Best Research for Best Health *[21], built on the previous phases and additionally positioned patients as the ultimate consumers of health research, leading to the establishment of the National Institute for Health Research (NIHR) [9].

4. *Including industry and other interests in a comprehensive overview of English health R&D*. The Cooksey Review was initiated by the Treasury almost immediately after implementation of the *Best Research for Best Health *reforms had begun [58]. The review examined the whole publicly funded system of health R&D in the UK, including the activities of the DH/NHS R&D programme and of the MRC where the traditional view of the independence of researchers is strongest. The review aimed to consider the needs of all customers, including some groups such as industry that hitherto had been less well represented.

Table 1 presents a timeline of key events in this history. The main element of each of the four phases listed above is shown in bold with the subsequent events constituting the main developments during that phase, or the key developments that drove the subsequent phase. For the final two phases, however, the main events run concurrently because the establishment of the NIHR was followed so rapidly by the Cooksey Review, which covered a broader spectrum of research.

**Table 1:** Timeline of four phases of reform and main developments in English health research system since 1970

Date	Title or name of key document or reform
**1971**	***Rothschild Report: The Organisation and Management of Government R&D: *** Led to government departments such as the health department receiving some of the funds of research councils and becoming the customers for research conducted by their contractors.

1973	Concordat signed transferring some Medical Research Council funds to the English Department of Health's research division. Consultative committee structure created for policymakers and researchers.

1978	Rothschild reforms such as consultative committees began to be dismantled.

1988	House of Lords Select Committee on Science and Technology: *Priorities in Medical Research*.

**1991**	***NHS R&D Programme: launched by Michael Peckham, first health department Director of R&D:*** R&D programme established to meet the needs of NHS.

1992	UK Cochrane Centre established as part of the NHS R&D Programme's information systems' strategy. This inspired the international Cochrane Collaboration.

1993	NHS Health Technology Assessment programme established.

1994	Culyer Report made recommendations to increase the accountability and transparency of research funding in the NHS and to protect the major research and teaching hospitals

1999	NHS National Institute for Clinical Excellence (NICE) established.

2002	*Clinical Academic Medicine in Jeopardy *Report published by the Academy of Medical Sciences to highlight the fears that academic medicine was becoming seen as a less attractive career

2004	UK Clinical Research Collaboration (UKCRC) created by the English Department of Health and many other stakeholders to enhance clinical research

2004	Final report from *Research for Patient Benefit *Working Party endorsed the creation of the UKCRC as a key part of developing a clinical research infrastructure embedded in the NHS,

**2005**	***'Best Research for Best Health' consultative document led to a series of reforms introduced between 2006-9:*** Comprehensive set of proposals aimed at meeting the needs of patients and other stakeholders

2006	*Best Research for Best Health *strategy document outlined the reforms to be introduced following the consultation to create the National Institute for Health Research (NIHR). Key features included:: building on the developing clinical research networks, raising the status of clinical researchers, introducing new funding programmes and centres to complement existing programmes such HTA programme.

2007	The first (of now 12) Biomedical Research Centres announced to support a critical mass of leading researchers in NHS/university partnerships that are driving innovation

2008	First 100 members of new NIHR College of Senior Investigators appointed thus raising status of clinical researchers and academic medicine

2009	NIHR Progress Report described how the *Best Research for Best Health *strategy is being comprehensively delivered by working across the NHS and with patients, health research academic institutions and industry.

**2006**	***Cooksey Review: *** Review of all publicly funded health research: endorsed *Best Research for Best Health *reforms, emphasised industry's needs and the importance of translational research, recommended creation of Office of Strategic Coordination of Health Research (OSCHR).

2007	OSCHR established as recommended. It integrated public expenditure bids from the English Department of Health (for the NIHR) and the business department (for the MRC) and, based on their success, achieved a record funding increase for health research. A Translational Medicine Board set up to work with MRC and NIHR to develop a fully aligned approach in translational research.

### The Rothschild reforms: government as consumer of research

In the 1970s, a major phase of R&D reform in England followed the 1971 Rothschild Report [56]. Health research system reforms in this phase were designed, in particular, to produce knowledge that would help policymakers in central government departments, including those responsible for health and social services policy. The Rothschild reforms envisaged government as the 'customer' for research, and the scientific community as the 'contractors', and described the relationship as being, '*the customer says what he wants, the contractor does it, if he can, and the customer pays'*. [56]. Many leading biomedical researchers opposed the resultant transfer of some research funds from the Medical Research Council to the health department which occurred through a concordat signed in 1973. Further, the use of the terms 'contractor' and 'customer' in relation to research was contentious at the time (and has continued to be so for some commentators [59]). Critics of the Rothschild principle have long claimed that it threatens the independence of researchers because it means the customer can determine the nature of the work to be undertaken in Government-funded research. Critics also dislike the deliberate incorporation of language from industry and commerce into academic research.

The English health department, which, as noted earlier, was then called the Department of Health and Social Security (DHSS), commissioned a seven-year formative evaluation of the reforms. This evaluation showed the difficulties in getting two very different systems - science and government - to work together. In their analysis, Kogan and Henkel highlighted the diversity of imperatives, tasks, approaches, and roles within and between each system [11,12]. Government rarely found it possible routinely to devote the time and resources to being an informed customer. Furthermore, for institutional and epistemological reasons, scientists often struggled to produce the knowledge needed by policymakers and, in turn, policymakers faced difficulties in acting as 'receptors' who could receive and use that knowledge. For much of the research commissioned, the Department was not even acting as the primary customer, but, rather, was in the position of a secondary or proxy customer for the field authorities and practitioners of the NHS who might have benefitted from research commissioned by the Department. This highlights the difficulties involved in attempting to identify, let alone meet, the needs of all stakeholders.

Kogan and Henkel identified that mechanisms at the boundaries - later called interfaces - between the research and policy systems, needed to be further developed in order to enable scientists and policymakers to work collaboratively, whilst taking into account their differences [11,12]. Such mechanisms included the Research Liaison Groups established in the 1970s, which attempted to bring together researchers, policymakers, professionals and research managers to identify relevant research topics in selected research areas. These mechanisms were of variable success, but some continued working well many years after other elements of the Rothschild reforms had been dismantled [11,12]. In addition to the variable success of those Research Liaison Groups that were created, there were quite a few topics which did not have their own group. In addition, and through its Policy Research Programme, the Department also had 'core'-funded Research Units (on rolling five-year contracts) and long-term programmes, some predating the Rothschild reforms. They, and similar units later funded by Regional NHS R&D Programmes, played an important role including developing collaborative research agendas [60-62]. Research Liaison Officers, who were recruited by the Department during this period (and are still a key part of the DH's research management today), helped such agenda building by acting as a 'hinge' between the people in the Department who took the lead on making policy and the health research community. In doing so they were fulfilling part of the 'brokerage role' identified by Kogan and Henkel as being a key mechanism [11]. The Nursing Research Unit at King's College, University of London, is an example of a unit from that time that received core, or long-term rolling, funding. It is still thriving and, at the time of its recent change of name to become the National Nursing Research Unit, policymakers continued to be able to describe its importance to them [63]. Its Research Liaison Officer continues to play an important brokerage role in linking the unit with the relevant parts of the Department.

The programme of reviews of the Policy Research Programme core-funded units conducted from 1979 also highlighted some of the difficulties that could be faced by scientists working on policy-related topics. When the main criteria used for the review of such units were those of traditional 'scientific merit', with a later separate review of policy relevance, this was perceived to have:

threatened the basic assumptions of some research units. Such units, often with the encouragement of the DHSS, concentrated on policy-relevant research that made no pretensions to influence the course of science. They feared that their work would be subjected to criteria quite different from those prevailing when they began it [12].

Though some institutional developments from the Rothschild reforms remained, many aspects of the reforms, including the transfer of funds from the MRC and the elaborate consultative committee structure, began to be dismantled from 1978 and did not survive the 1980s. This was partly because of the continuing opposition to the customer-contractor principle from significant parts of the medical research community, and partly because of the difficulties faced by the Department in acting as an informed customer [11]. Even for policymakers dealing with issues which were the responsibility of the central department, the role of customer proved to be more difficult than anticipated - partly because of the time required.

### NHS R&D programme/Peckham reforms: the NHS as consumer of research

As a result of the difficulties in implementing the Rothschild reforms, many of the issues related to addressing the needs of policy costumers were not fully resolved. There was increasing concern in the UK in the 1980s that the health research system was still neither properly serving the needs of the health system itself [64], nor those of the Department of Health, and, perhaps partly as a result, that the NHS was not adequately meeting the health needs of the country. In 1988, an influential Parliamentary review was published by the House of Lords Select Committee on Science and Technology [13]. Its main recommendation was that health research should give greater emphasis to addressing the needs of the NHS, seen as a research 'customer' in its own right but with an insufficient voice hitherto [13].

While some reservations were expressed about whether there would be sufficient organisational capacity and resources to undertake the wide range of tasks required [60], and not all the proposals from the House of Lords Committee were implemented (including the idea of a National Health Research Authority), many of the key ideas behind the report were introduced in a major phase of reform began under the first Director of R&D, Michael Peckham. The detailed strategy for this phase was set out in 1991 in *Research for Health: A Research and Development Strategy for the NHS *which claimed that the new programme 'presents the opportunity of working towards a coherent national programme of research in collaboration with the MRC, the charities and industry' [17]. To help achieve this strategy it was intended that over a period of five years there would be an increase of approximately 50% in the budget of the new NHS R&D programme so that it reached about 1.5% of the NHS budget. The main focus of the strategy, which applied in England, was an attempt to integrate the new NHS R&D programme into the management structure of the health care system. This required a major effort; no other country had ever attempted such an ambitious approach [14,65]. A range of issues was addressed, including attempts to commission research to meet the needs of the health care system, attempts to encourage the greater utilisation of research within the health care system, and various changes in organisation, management and governance of research. A key role was to be played in this by the NHS regions within England:

The regions will have a crucial role in the R&D programme - helping to shape the overall strategy, setting their own priorities, directing, commissioning and managing research and development programmes and helping to ensure that the results of good research are used to full effect [17].

However, although using the NHS regional structure did facilitate attempts to build the R&D process more closely into the customer organisation, it also made the R&D system vulnerable to changes in the wider NHS system. The regional dimension made a useful contribution to a range of activities such as agenda-setting and encouraging the appreciation and use of research, for example, as noted above, through the provision of critical appraisal of research evidence courses [39]. But when the regional structure of the NHS itself was eventually amended, and later disbanded, reorganisation of the R&D structure was also necessary [12].

In aiming to meet the needs of the NHS, another major element of the new strategy was the creation of a series of time-limited national NHS R&D programmes [66]. These covered topics that had been identified as priorities for the NHS. Most related to specific fields such as the management of asthma, cardiovascular disease and mental health, but some were cross-cutting fields, including the Implementation Methods Programme described below [50]. A key feature of these programmes was the involvement of a wide range of stakeholders, including patient representatives, on both Advisory Groups that identified R&D priorities and on Commissioning Groups that decided which proposals to fund as part of each programme [50].

In addition to the time-limited programmes, in 1993 a permanent Health Technology Assessment (HTA) programme was established following a report that had emphasised its importance to the NHS [67]. This continues to the present and has become increasingly successful in allowing various parts of the health care system to become more directly involved in setting the research agenda relating to the assessment of specific treatments, drugs and devices [55]. Furthermore, it is now 'internationally acclaimed' [58], and its research is much used by various policymaking bodies, including those described above, ie the National Institute for Health and Clinical Excellence (NICE) and the National Screening Committee (NSC) [55]. Indeed, NICE was explicitly established in 1999 to serve as a 'receptor' organisation whose work to inform clinical and commissioning decisions in the NHS would be directly informed by HTA and other clinical research [12]. The existence of bodies such as NICE and the NSC gives authority to some HTAs, thus enhancing the status of the knowledge production involved in this type of research, which is important if its impact is to be sustained [12]. NICE guidance documents have become increasingly binding on the English NHS. Each one is informed by a research report specifically commissioned from the HTA Programme, and NICE also issues advisory clinical guidelines, which are informed more broadly by the portfolio of work produced by the HTA and similar programmes. In general, bodies such as NICE and NSC act as receptors for a wide body of research conducted in the English health research system.

Even before the creation of NICE, there were determined efforts by the health research system to address the problems of under-utilisation of research within the National Health Service. These included a research information systems strategy which provided funding for the establishment in 1992 of the path-breaking UK Cochrane Centre [66] that subsequently inspired the international Cochrane Collaboration. The strategy also led to the creation of the NHS Centre for Reviews and Dissemination at York University, primarily to undertake synthesis of the findings from clinical research. In addition, in the mid-1990s, the NHS R&D programme launched the Implementation Methods Programme which funded pioneering research into methods to implement research in clinical practice [68]. This was probably the first research programme in the world specifically focused on methods for implementing health research and was a further important element in the attempt to make the then NHS R&D system serve the needs of the National Health Service [50]. Overall, the information systems strategy has been one of the most enduring parts of the Peckham reforms and has had a major international impact. Whilst progress in terms of greater utilisation of the research has been more difficult, in the UK it was recognised that in some areas considerable progress was made on the uptake of effective, research-based clinical practice, for example in maternity care [69].

Since at least 1990, consumers and the public have been involved in different aspects of R&D related to the NHS [46,47]. In one study published in 2001, 42% of NHS providers reported that they had involved their patients in some way in their R&D activities, but there was some confusion about what this entailed and a lack of awareness about NHS performance indicators for consumer involvement [46]. An additional element of the NHS R&D Programme was established in the mid-1990s to make progress in this field. Originally called 'Consumers in NHS Research', it continues to this day under the name of INVOLVE, and aims to ensure, 'that people's involvement in R&D improves the way that research is prioritised, commissioned, undertaken and disseminated' [70]. As with various other issues discussed in this section, some of the most innovative developments in consumer involvement came at regional level within the NHS. One example of an attempt to facilitate the role of the patients and the public was the use of innovative virtual research commissioning panels by the R&D Directorate of the former London Regional Office of the DH [49].

Given the additional resources that were being allocated to the NHS R&D programme it became increasingly important to attempt to demonstrate that wider impacts or benefits were flowing from the research commissioned. Therefore, the Department of Health commissioned a stream of work that resulted in the Payback Framework for categorising and assessing the wider impacts made by health research [71]. The framework consists of a multi-dimensional categorisation of benefits and a logic model to help organise the assessment of the impact. The Payback Framework was used in guidance given to directors of DH 'core'-funded units and programmes about preparing for their DH review: it was seen as an approach that could be used by the units to demonstrate the wider impacts that their research was making on policy and health care within the NHS [12].

In 1996 another programme appeared in official papers and was formally launched in 2000 as the NHS R&D Service Delivery and Organisation Programme [15]. It had a remit that, at least as viewed later, seemed to stretch broadly to include specific organisational issues of concern to managers as well as key policy issues of concern to the NHS. There was a potential overlap here with the direct concerns of the Department of Health, whose centrally commissioned research programme continued to meet its own research needs throughout the Peckham reforms. Despite all these positive changes, some of the difficulties with organising and assessing policy-related research that had been highlighted by Kogan and Henkel remained unresolved. In a 2003 analysis that perhaps downplayed the progress that had been made with initiatives such as the HTA programme, a report for The Health Foundation and the Nuffield Trust identified many barriers to health services research supporting the improvement of health care services in the UK [32]. The report claimed that these barriers included: a lack of research into prime areas of importance for policymakers, clinicians and managers of clinical services; the frequent inaccessibility of the research that was conducted; and the fact that the reward structure of the UK's Research Assessment Exercise (a method used to assess the performance of university research in the UK with attendant implications for the distribution of government funding to universities) did not encourage research relevant to improving the health care service. Some of the recommendations made in this report echoed the earlier analysis from Kogan and Henkel [11]. Furthermore, others argued that major aspects of the reforms called for by the House of Lords report, such as an expansion of public health research, had not been sufficiently implemented [72].

Another issue that proved to be problematic was the large amount of continuing NHS R&D funding provided to meet the NHS costs of hosting research supported by eligible external funders, such as the MRC and Wellcome Trust. Attempting to improve the management and coordination of this element of the NHS R&D budget was a major task throughout this period. Whilst the long battle to increase transparency and accountability of this money is described in detail elsewhere [15], it is worth noting that changes in the NHS itself increasingly impinged on the R&D system that was trying to meet its needs. Changes such as the purchaser/provider split meant that the major research hospitals faced the potential danger of becoming seen as inefficient health care providers because of their research and research support costs. To address these increasingly pressing concerns, the Culyer review was established in 1994 and the NHS R&D funding streams were reorganised to protect the research and teaching hospitals [73]. Various reforms followed but, even in the early 2000s, concerns were still being expressed about the level of transparency in using the £400 million that was spent annually on meeting the NHS costs of the MRC and charity funded R&D [15].

In the early 2000s, further problems, largely unconnected to the Peckham reforms, began to emerge, including those facing medical academics, as described in 2002 in the report from the Academy of Medical Sciences entitled, *Clinical Academic Medicine in Jeopardy *[74]. This suggested that increasing service pressures on clinical academics reduced their time for teaching and research when other resources were also declining: for example, it was claimed that the Research Assessment Exercise had also resulted in falling investment in technical support staff. As a result of all these pressures, clinical academic medicine was no longer seen as an attractive career [74]. The next year two more reports built on this and reflected the views of a range of stakeholders: the first was a further report from the Academy of Medical Sciences [75], and the second a report from the Biotechnology Innovation and Growth Team [76]. The two reports identified a series of critical challenges including inadequate support for clinical research. In the light of this, the *Research for Patient Benefit *Working Party was established with the remit to 'bring forward to ministers practical proposals for implementing the recommendations in the two reports' [77]. An interim report examined the components that the two reports had recommended were necessary to boost clinical science, including the development of a clinical research infrastructure embedded in the NHS, and concluded that government and charitable funders had made some progress in those areas but it was by no means sufficient in depth or breadth. The final report, published in April 2004, endorsed the creation of the UK Clinical Research Collaboration to encourage the engagement of a wide range of stakeholders, and stated that it 'should adopt as its long-term goal establishing the NHS as the world leader in contributions to clinical research.' [77]. These developments helped prepare the way for the next set of reforms.

### The 'Best Research for Best Health' Report and the National Institute for Health Research: multiple stakeholders including patients as consumers of research

In 2005, a further phase of health R&D reform was proposed in the *Best Research for Best Health *consultative report [21] and, following consultation, implementation of its recommendations began in 2006 with the creation of the National Institute for Health Research (NIHR), led by the Director of R&D in the Department of Health [9]. These changes re-emphasised the role of research in addressing the needs of policymakers, managers and health service providers, but the reforms additionally presented patients and the general public as the ultimate consumers or beneficiaries of health research. This was in line with developing trends elsewhere in health policy. Several reports had increasingly demanded that health research should focus on the needs of patients and the general public [15]. As noted, the *Research for Patient Benefit *Working Party had been established and made recommendations for boosting clinical research in the NHS. The INVOLVE programme had also been established.

Whilst the *Best Research for Best Health *consultative document gave considerable emphasis to meeting the needs of patients, the majority of the eventual reforms concentrated on the structure and organisation of the health research system and on ensuring that more clinical research was to be conducted. The aim of the consultation was to 'draw out any issues stakeholders may have had with regard to the strategy, and to inform our final proposals' [21]. The overall direction of the proposed new strategy received considerable support during the consultation, but there were inevitably some areas where concerns were raised. These included:

• fears about whether concentration of excellence in proposed new clinical research centres would drain the rest of the NHS of talent and research opportunities;

• concerns that primary care research and non-clinical disciplines might not receive sufficient consideration;

• a view that greater weight should be given to the resource represented by patients and the public; and

• the view that it was important to try to assess the impacts of the changes in terms of improved health care outcomes.

The NIHR was established to provide coherence, focus and status to the different strands of research covered by NHS R&D and DH funding [9]. In some ways, the proposal for such an Institute reflected the unimplemented recommendation of the 1988 House of Lords Report in support of a National Health Research Authority [12,13]. The outstanding feature of *Best Research for Best Health *is probably the breadth of the strategy, as highlighted by Sally Davies, Director of R&D in the DH, in her postscript to the final strategy document published in 2006 following the consultation: 'we want to emphasise that the strategy does not consist of one or two 'big ideas' in isolation' [9].

Given the wide-ranging nature of the *Best Research for Best Health *strategy, it is impossible here to describe all its elements. Progress on the full range of activities is described in the NIHR's own reviews of progress [78,79] and in the regularly updated implementation plans available on the NIHR web site [80]. Reflecting the government's desire for a still more comprehensive approach, the Cooksey Review of the wider English health research system began shortly after the *Best Research for Best Health *strategy began to be implemented. However, the *Best Research for Best Health *strategy has continued because the implementation of the recommendations from the Cooksey Review complements that strategy rather than instituting a different structure. Therefore, in this section we shall describe the implementation of *Best Research for Best Health *to date before going on to describe the wider reforms that resulted from the Cooksey Review and were introduced to run concurrently.

A key feature of the NIHR reforms is that there have been a series of developments, not all of which were fully set out at the start of the process in 2005, but all of which have come under the *Best Research for Best Health *umbrella. Therefore, rather than attempt to list all the developments, the account below highlights some of the key developments as they relate, in particular, to the needs of the stakeholder groups described above. Some of the major themes addressed, therefore, concern how the strategy attempts to bring various stakeholders together, how some of the problems facing medical academics are being tackled, and how the diverse funding streams meet a variety of needs of the different stakeholders.

Turning to the first of these major themes, the *Best Research for Best Health *strategy recognises the importance of involving a wide range of stakeholders, including industry and the medical research charities, in identifying R&D priorities and enhancing the utilisation of research. Key features of the reforms include building on the research networks that were beginning to emerge at the end of the previous phase, and especially on the UK Clinical Research Collaboration created by the DH in 2004 in partnership with other research funders, industry, regulatory bodies, Royal Colleges, patient groups and academia [81]. One of the striking features of the UK Clinical Research Collaboration mission set out in 2006 refers to the importance of meeting the needs of industry by involving it in agenda-setting for health research within the NIHR and by providing a suitable location within the NHS for companies to fund trials of their new drugs and devices [81].

The UK Clinical Research Network was established to complement the work of the UK Clinical Research Collaboration. This network consists of a managed set of topic-specific clinical research networks (which at the time of the 2005 consultation already covered cancer and mental health), a Primary Care Research Network and the NIHR Comprehensive Clinical Research Network, created to provide a world-class infrastructure for clinical trials in all areas of disease and clinical need within the NHS [82]. Each network is built up of regionally based Local Research Networks, and by 2009 there were 25 NIHR Comprehensive Local Research Networks. The networks are not only intended to remove barriers to the support of public and charitable funded research by the NHS and increase the number of people who enter multi-centre trials, but also 'ensure that the NHS can meet the health research needs of industry' [9]. The networks therefore support trials funded by the NIHR itself, and those funded by 'other partners including the Medical Research Council, medical charities, such as the Wellome Trust, and the life sciences industries.' [79]. The various networks are also designed to ensure that the needs of industry are met to a greater extent than previously. The NIHR progress report for 2008/9 also gives examples of where a Comprehensive Local Research Network helped fund posts to enable the professionals in local NHS trusts undertake clinical trials. As noted above, a major reason behind the creation of the networks was to tackle some of the problems facing clinical academics.

The feeling that there was a lack of incentives for able doctors to become medical academics was a major driver behind the *Best Research for Best Health *consultative document. It described disincentives to entry and barriers to progression. In an attempt to address some of these problems reforms are being introduced by the NIHR, including: raising the status of researchers through badging them as 'NIHR faculty' and ensuring their funding is separate from the NHS's patient care budget; expanding capacity development programmes for future leaders in applied health research; and working with UK Clinical Research Collaboration partners to create additional training opportunities for clinical academic posts [9]. In relation to the creation of the NIHR, Shergold and Grant wrote, 'From an historical perspective, the creation of this outspokenly competitive new body represents the Department's boldest step yet to exorcise the one time stigma of its researchers as "second-class scientific citizens"' [10]. An element in achieving this is the appointment of researchers to a College of NIHR Senior Investigators. According to the latest progress report:

this prestigious three-to five-year award enables us to harness the skills and knowledge of the country's most prominent clinical and applied health researchers to tackle the challenges facing the NHS. They are chosen by rigorous peer review for their outstanding achievements as the best researchers in their respective fields. [79]

It remains to be seen how this will develop and, indeed, the system does depend on researchers identifying themselves as the most prominent and applying to join. A further crucial departure from the earlier periods is the recognition that the incentives for applied/health services researchers, and how their work is assessed, must match their tasks and research objectives. This was explicitly recognised both in the NIHR strategy and in the creation of the independent Health Services Research Network by the research community itself which is beginning to give greater recognition to the role of this type of research. In relation to the 2008 Research Assessment Exercise, the Department of Health and NIHR successfully helped promote the idea that the wider impact of research should be taken into account where relevant. This has helped stimulate further work developing ways of assessing the wider impact of health research on health policy [83].

To meet a range of objectives and the needs of various stakeholders, the *Best Research for Best Health *strategy proposed to introduce a raft of new funding streams for projects, programmes, units and centres, in addition to additional funding for existing programmes such as the HTA and Service Delivery and Organisation programmes. Progress was being made on these funding streams when the Cooksey Review, described in the next section, highlighted the importance of addressing the two translational 'gaps' in the production and application of research and described the critical pathway required to turn biomedical research developments into health benefits for patients [58]. The framework provided by Cooksey provided a structure for the NIHR to develop: both subsequent NIHR progress reports set out how its funding schemes fit into, and, in effect, constitute, a major part of the 'innovation pathway' described in the Cooksey Review [78].

The success of the NIHR HTA programme was noted above [55,58], and it illustrates the way in which the Cooksey Review has enhanced the NIHR. The NIHR HTA Programme has received increased funding resulting from the *Best Research for Best Health *strategy and then further increases following the Cooksey Review [58]. The HTA Programme is not only playing an increasingly important role in informing the decisions of policy makers and managers in the NHS, but it has also taken steps to introduce the recommendations of a report on how to improve patient and public involvement in setting its research agenda [84].

The NIHR Service Delivery and Organisation R&D programme provides research for a broad range of potential users in the health care system. Following the establishment of the NIHR in 2006, the mission of the Service Delivery and Organisation programme has shifted to focus more on producing evidence that improves practice in relation to the organisation and delivery of health care and less on the policy-related research that is the responsibility of the DH's Policy Research Programme [85]. As noted above, this neat distinction is not, however, clear cut in practice. Compared with the HTA programme which is largely addressing the needs of clinicians, it is also generally more challenging for the Service Delivery and Organisation programme to identify and meet the needs of managerial stakeholders since they are harder to identify [32]. Nevertheless, its research too is beginning to be used by organisations within the health care system [86] and current initiatives are aimed at enhancing the capacity of the programme to meet the needs of that system [87]. Since its establishment the Service Delivery and Organisation programme has made considerable efforts to demonstrate that a wide range of methods should be seen as appropriate for the type of the research it commissions [88]. More recently the NIHR Service Delivery and Organisation programme has also collaborated with the Canadian Health Services Research Foundation to review methods for synthesising qualitative and quantitative, and 'mixed' method research in ways that are useful for health policymaking and management [89-91].

The HTA programme and the Service Delivery and Organisation programme are parts of the health research system that are seen to have a key role in achieving the increased public participation in health issues (and not just in research) called for in the 2002 Wanless Report [92], which considered the long-term resource requirements for the NHS in the UK. Wanless set out three scenarios related to the degree of public involvement in health matters. Of the three, the 'fully engaged' scenario would achieve maximum improvements in health status at lowest cost. In reviewing progress, Wanless concluded in 2007 that the population was a long way short of being 'fully engaged'[93]. Wanless referred to the role of the Service Delivery and Organisation programme and the Cooksey Review also concluded that an expansion of the HTA programme would be crucial to the delivery of Wanless' 'fully engaged' scenario.

The new streams of research funded by the NIHR include the responsive mode, Research for Patient Benefit Programme, which 'allows health service professionals themselves to come up with ideas with the potential to improve their everyday practice' [79]. A School for Primary Care Research has been created, and further recent developments within the NIHR include the launching of two new programmes. First, a Public Health Research programme aimed at providing information to enable public health interventions delivered outside the NHS and health care settings to be based on sound evidence. Second, a Health Services Research Programme to fund studies into better ways of planning and providing health services, including, for example, studies of cultural and organisational issues affecting patient safety [79]. These are areas which had risked being overlooked.

2007 saw the start of one of the major initiatives introduced as part of the NIHR, ie the creation of what are now 12 Biomedical Research Centres, on which £117 million is being spent in 2009/10, and 17 Biomedical Research Units on which over £20 million a year is being spent [79]. These University/NHS-based Centres and Units address several of the issues of concern in relation to academic medicine and clinical research highlighted previously. They each support a critical mass of leading researchers in NHS/university partnerships that are driving innovation in the prevention, diagnosis and treatment of ill-health, and are translating advances in biomedical research into patient benefits. Furthermore, the Centres and Units provide enhanced support for those wishing to pursue a career as a medical academic, they have growing links with industry, and they are fostering new levels of cooperation between researchers from different disciplines, between researchers and the NHS, and also between researchers and patients. Most recently, some of these Centres, and in some cases associated Units, have been designated by the Department of Health as Academic Health Science Centres. The five so designated by 2009 do not receive any additional funding as a result, but it has already become a much coveted status. However, even among those who recognise the potential advantages in concentrating considerable resources on the new Biomedical Research Centres, there is the concern that it should not be at the expense of less research-intensive universities and hospitals [94].

Indeed, in the original 2006 strategy document, it was claimed that the NIHR 'does not aim to be either elitist or egalitarian' [9] and as well as concentration in the leading centres some of the developments have tried to encourage a wider distribution of some funding to good researchers throughout the NHS. For example, the Collaborations for Leadership in Applied Health Research and Care (CLAHRCs), have been developed in response to the Cooksey Review and are aimed at funding partnerships between medical schools in various parts of the country and local NHS organisations to develop innovative ways in which health research can be conducted to improve the effectiveness of clinical care [78]. This goes some way to address the fears about a narrow concentration of research funding within parts of the NHS.

Looking at the current strategy of the NIHR in the context of the earlier phases, it is possible to see it as one of continuing the integration of the health research system more closely into the Health Service, and yet at the same time attempting to provide greater financial independence and security for the research system. Writing a postscript to the 2009 NIHR progress report, Sally Davies, now Director General of Research and Development at the DH states:

When we first launched the NIHR, we...could see the huge potential for improving, expanding and strengthening the way that health research is delivered for patients, the public and the NHS. What we had to do was create a paradigm shift in the national research environment and we gave ourselves three short years in which to achieve the transition. Well, we have now passed out of transition, and it is true to say that the NIHR has really taken off [79].

### The Cooksey Review and the Office for Strategic Coordination of Health Research reforms: including industry and other stakeholders

Shortly after implementation of the new National Institute for Health Research had begun in 2006, the UK Government announced that the Treasury would conduct a further review of the wider UK medical research field, taking into account the recent decision to create a single health research budget amalgamating the research budgets of the MRC and the DH. The resulting Cooksey Review recommended that a new overarching body be created to oversee the single research fund: the Office for Strategic Coordination of Health Research [58].

Whilst the three previous phases of reform were primarily attempts to cater better for specific stakeholders whose needs were perceived as not being adequately met, the potential strength of the Cooksey Review, and the resulting Office for Strategic Coordination of Health Research, is that the system has been considered as a whole, including the more basic biomedical research funded by the MRC and the major medical research charities. The review endorsed the *Best Research for Best Health *reforms, and called for structures to encourage the coordination of the system, including by building on the efforts of NIHR to structure and formalise the growing focus on networking between the various stakeholders in the health research system. What was particularly challenging about the Cooksey Review, perhaps not surprisingly given its Treasury origin, was the extent to which it emphasised the potential contribution of health research to the UK economy. The opening paragraph of Cooksey's Review stated:

the Government's vision is of a holistic health R&D system that will maximise the value of the UK's health research base, ensuring the UK's health research is more closely aligned with wider health objectives, builds on scientific progress to date, and translates the results of research into economic benefits [58].

The concerns of industry were prominent in the conclusions and recommendations of the Cooksey review [95]. A key goal, and the one that concludes the foreword to the report, is that the UK should be, 'an outstanding location for healthcare companies to develop their business' [58]. The Review proposed that government, regulators and industry should create a new partnership 'to pilot a new drug development "pathway" to create winners for all stakeholders: industry, government, the wider economy and, most importantly, patients'. One of the key issues was that the pathway should streamline the processes involved in setting up and paying for clinical trials in the UK.

The underlying theme behind the Cooksey Review was to ensure that the whole process of knowledge transfer from 'bench to bedside' worked better. In particular, it highlighted two translational 'gaps': the need to improve translation from the laboratory to the creation of new products and treatments; and then the need for translation of those new products and approaches into routine clinical practice [58]. Cooksey made a series of specific recommendations for how parts of the overall publicly-funded health research system should collaborate with industry to tackle these two translation gaps. To address the first gap, he proposed that the Office for Strategic Coordination of Health Research (OSCHR) and a new Translational Medicine Funding Board work with the health care industries and other stakeholders to develop proposals for joint private and public investment in new technologies for medicines' discovery. A Translational Medicine Board has now been established and its role is 'to work with the OSCHR Partners to develop a coordinated, coherent, fully aligned research strategy in translational research.' [96]. Specifically to address the second gap, Cooksey proposed that the NIHR HTA Programme and NICE should work with industry to identify new medicines under development that might be suitable for earlier HTA and earlier NICE guidance decisions [58].

As we have seen, by endorsing the NIHR, the review helped facilitate the implementation of the *Best Research for Best Health *reforms, and in many ways strengthened them. The Cooksey Report called for better training for NHS managers and clinical staff to improve their understanding of the benefits of research and noted that the Canadian experience provided an example of how it might be done [58]. Also in Canada, the review noted, additional funding to underpin the changes needed at the time of the establishment of the Canadian Institutes of Health Research was widely seen as having been of vital importance to the success of the initiative. One of the Office of Strategic Coordination of Health Research's key roles is to make a single submission for health research funding to the Treasury on behalf of the two government departments responsible for health research [96]. In its 2007 submission to the Treasury it highlighted the success of the NIHR and MRC and outlined an ambitious programme of coordinated work to enhance translational research; in October 2007 it secured the largest ever increase in public funds for UK health research [96]. This increase is intended to support the areas identified in the Cooksey report as needing additional funding, including evaluation and trials to ensure that so called 'basic' science is translated into health and economic benefits for the UK, and public health research. Crucially, the increase in funding for applied research for 'patient benefit' has this time, unlike in the 1970s, come in the form of additional funding for health research and not as a switch in priorities away from basic biomedical science. The focus on the publicly-funded health research system as a whole, and its relationship to other stakeholders, followed by the establishment of the Office of Strategic Coordination of Health Research, have all been steps towards the creation of a national health research system.

The Cooksey Review has been seen as 'a masterful attempt at coherence' [95], and whilst its success is still not guaranteed, nevertheless, an early assessment of these reforms in the *BMJ *in February 2008 was cautiously optimistic:

So could this prove to be a reorganisation prompted by a correct diagnosis and followed by a prescription of the appropriate remedy? The final verdict will come later. For the moment though that is more or less how it looks [97]

## Lessons for building a health research system

Building any health research system to meet the needs of diverse stakeholders is complex. Here we present some general lessons from the analysis about how diverse needs can best be met, and at each stage make some observations about how far the reforms in English system have met stakeholder needs. We also discuss recommendations for both the English system and more generally.

### 1. Facilitating overall and ongoing coordination

Kogan and Henkel's analysis, in particular, highlighted that the many elements in a system of health research create tensions between stakeholders as different people represent different interests and fulfil different roles, with accompanying differences in values [12]. As we have stated, the key stakeholders include health care policymakers, health care providers, patients, industry, and, of course, researchers themselves. The needs of these five groups are summarised in Figure 1. The first lesson, therefore, is the importance of addressing as coherently as possible the diverse needs of the institutions and actors that interact in any health research system and recognising that a degree of coordination will be necessary. But it goes further than this: there is a need to develop appropriate structures (such as the new NHS/university collaborations being developed by NIHR), build cultures, and fully and effectively use developing technologies such as systematic reviews. It is important to note that to meet the needs of all the stakeholders it is likely that a full range of research should be undertaken, including Mode 1 and Mode 2 research as described in the Introduction.

In promoting this coordination, research managers can play an important role by strengthening the interfaces between various stakeholder groups. This role is often overlooked and consequently under-resourced [12]. The main things that research managers need from the health research system are recognition of their role (in all its complexity) and adequate resourcing of research management tasks. In order to be able undertake their roles, research managers need a range of capacities [11,12], including being able to: establish and manage some of the research interface mechanisms described below; communicate and broker different R&D needs and findings; and identify and resolve gaps and conflicts between groups, and synthesise research perspectives.

More generally, it is important to realise that in complex systems there are always new challenges arising and new perspectives developing both as a result of continuing consultation and as a result of new research into various aspects of the system. This puts a premium on the system being flexible enough to adapt to developing thinking, whilst being sufficiently robust to maintain the key elements. In organising and running the health research system, it is appropriate to keep in mind the more general lesson proposed by Alan Irwin about the importance for the relationship between science and society of 'the development of an open and critical discussion between researchers, policy-makers and citizens' [5].

Looking specifically at the system in England, throughout the last four decades we have seen some continuities in terms of developments that have lasted, for example, the idea of having applied research units focused on specific needs of the health care system. We have also seen other examples of reversals. But, and particularly over the last two decades, many of the initial advances have consolidated as those running the health research system, often working closely with those running the health care system, have increasingly attempted to analyse and reform the system as a whole. Examples of where this has worked well include the HTA programme established as early as 1993 to undertake research explicitly to meet the needs of the NHS. Its role and importance increased with the establishment in 1999 of NICE which has acted as a key 'receptor' body for much of its research. Its role expanded subsequently and finally its role has expanded even more within the 'innovation pathway' set out by Cooksey. The key elements of the information systems strategy established in the 1990s, including the UK Cochrane Centre and the Centre for Reviews and Dissemination at the University of York, continue to be funded as core elements of the NIHR and have been hugely influential, both in the UK and elsewhere.

The NIHR, created in 2006, has gone further than previous arrangements in explicitly incorporating the needs of stakeholders such as industry, and addressing the growing problems that were facing medical academics. In general these developments seem to have been organised in a way that has not been obviously at the expense of existing key stakeholders. Patients and the public continue to be regarded as a key stakeholder group, and the initial steps taken in the 1990s, such as the introduction of patient representatives onto commissioning panels and the later creation of INVOLVE, have been built on, although to date it is difficult to assess the impact of progress on some of these issues [45].

Some of the initiatives in the 1990s, especially the creation of Regional R&D structures, were largely discontinued because of changes in the wider NHS. But subsequently the creation of the Local Clinical Research Networks, operating at a regional level, indicates that developments at this level are still seen as playing an important role in the success of the overall system. These developments should be less vulnerable to changes in specific NHS healthcare structures.

Probably the most striking feature of the reforms introduced since 2006 through the NIHR has been the attempt to integrate a range of initiatives into a NHS/DH system that builds on existing strengths, and operates with an effective level of coordination to meet the needs of various stakeholders. More recently, with the Cooksey Review, there have been largely successful attempts to develop a coordinated system for the whole of portfolio of publicly-funded health research and to do so in a way which also engages in effective liaison with stakeholders such as industry and the medical research charities. Three key features stand out here. First, Mode 1 and Mode 2 research are both supported and integrated into an overall system in the 'innovation pathway'. Second, the drive towards creating structures to foster effective translational research provides an overall rationale for many recent developments. Third, the increased public funding for health research during the lifetime of the NIHR has created the circumstances in which most stakeholders in the health research system have been in some way 'winners'. As discussed later, how sustainable this will be remains to be seen.

### 2. Need for interface mechanisms

A second lesson is that it is essential to develop, or strengthen, mechanisms at the interfaces between the health research system and different groups to address their needs, and to enhance understanding, communication and the use of research. Evidence about research management highlights the need to develop effective interfaces between researchers and policymakers, professionals and patients [4,11,16]. What is absolutely key here is that, unless there is acknowledgement of the specific needs and interests of different groups, and an analysis of the required interface or boundary tasks to meet these needs, any strategy risks under-achieving. Kogan and Henkel highlighted the many issues that arise at the boundaries between a research system and the wider society when the research system moves beyond concentrating on purely traditional academic concerns [12]. This approach was built on in the Payback Framework developed to assess the wider impacts of health research and in the 'interfaces and receptor' model [16,40]. A series of key interface or boundary tasks and activities can be identified for a health research system that is attempting to meet the needs of the health care system and the economy. They include:

• research problem and needs identification, and agenda setting (priorities);

• research commissioning in line with the identified priorities;

• reviewing and synthesising research;

• communicating findings to the health care system, patients and the wider society;

• facilitation of research absorption and utilisation in the health care system; and

• performance assessment and incentives for researchers working on applied research issues.

The first column of Table 2 illustrates the generic tasks at the interfaces, as discussed above. The second column illustrates some of the mechanisms that now exist in the English health research system to undertake these 'interface' tasks. It has been possible to develop a system that addresses many of the interface issues and, at least to some extent, allay the concerns listed above that were raised in the original consultation on *Best Research for Best Health*. But despite recent progress the third column shows there are still some areas in the English system that might potentially require more attention.

**Table 2:** Illustrative examples of interfaces/boundary tasks and mechanisms

Tasks & activities at the interfaces/boundaries between stakeholders	Examples of interface mechanisms in English Department of Health's R&D system	Areas that may require more attention
**Agenda setting/research problem definition**: Research funders scoping research needs and priorities, negotiation/consultation with the various stakeholders	The role of the Biomedical Research Centres in bringing many interests together; advisory groups e.g. HTA, for consumer involvement; NICE; various UK health research collaborations and clinical research networks that involve many stakeholders including industry.	Capacity to undertake collaborative comprehensive/systems needs assessment, especially for policy research in some non medical areas (eg workforce issues) where there has been less focus.

**Research commissioning**: Engaging stakeholders in research specification & selection of researchers	Diverse commissioning panels for the wide range of NIHR programmes, including HTA, SDO programmes.	Development of role of different actors (especially patients/public) and the need for innovative methods to involve them.

**Research processes**: Researcher control of methods but liaison between researchers & users during projects & collaboratively undertaking research	DH Research Liaison Officers; clinical research networks help to ensure research capacity is developed.	Researchers' ability to control methods when demands are made for speeding up of processes

**Reviewing & synthesising research**	HTA & SDO programmes; funding for UK Cochrane Centre and Centre for Reviews and Dissemination (CRD); DH Policy Research Programme projects.	Agreed upon methods and capacity for reviewing organisational and policy research; building on the SDO/Canadian initiative;

**Research communication**: Formatting research for different users; research brokerage; research networks	CRD; DH Research Liaison Officers; Cabinet Office Policy Hub; topic-specific research networks; integrating research information systems & databases.	Expanding research brokerage to link user groups at the systems level; developing systems-level media and communication strategy

**Facilitation of research absorption & utilisation**: Building capacity to receive and use research for policy, practice, and informed health decision-making	NICE; National Screening Committee; clinical research networks; NIHR Biomedical Research Centres and Units and Collaborations for Leadership in Applied Health Research and Care will lead adoption of research into clinical practice	Strengthening capacity, eg of receptor bodies for SDO and policy research; further developing absorptive capacity widely through NHS - geographic spread and the full range of staff; further improving population health literacy.

**Research performance assessment**	NIHR successfully argued for wider impacts of health research on policy and practice to be included in the Research Assessment Exercise as it is in the review of DH research units	Ensuring NHS performance measures reflect research contributions.

**Research incentives**: Appropriate for different types of research	Researchers (ie 'Faculty') becoming part of NIHR to attract clinical researchers; Senior Investigators important.	Further development of ways to recognise health services and policy research contribution to healthcare and the wider economy

Some of these are discussed below and a fuller list of points is summarised in Table 2:

A. In terms of problem identification and agenda setting, considerable progress has been made in allowing a range of stakeholders to contribute to identification of research topics, but a lesson from the Rothschild reforms is that it can require a major time commitment from policymakers and they may not always be willing to engage sufficiently with researchers. It is not entirely clear how much scope there is for collaborative development of research agendas on the major, and system-wide, policy areas, especially in some of the non-medical or technological areas (for example, issues to do with the education, careers and working conditions of health professionals) that have generally received less attention. Furthermore, the emphasis on translational research has also highlighted the need for greater collaboration between researchers from different backgrounds, for example, basic and clinical researchers. Attempting to get researchers from different backgrounds to work together on developing agendas is one of the issues being addressed by some of the recent developments in the NIHR, including Biomedical Research Centres and Units.

B. In relation to research commissioning there have been various successful initiatives to involve a wider range of stakeholders (for example, in the HTA programme), but despite widely available and regularly updated guidance from INVOLVE it is not yet clear that ways have been found to address all the difficulties that face patients and the public seeking to play a full role in research commissioning groups [45].

C. It is not clear how potential conflicts will be resolved between the pressures to speed up processes in translational research such as the production of HTA reports for NICE, and at the same time allow researchers to control the research process to ensure high quality.

D. The health research system in Canada has in some ways gone further than the English system in demonstrating the scope for developing both research 'brokerage' roles to link with users [98] and training programmes to develop the absorptive capacity for research in health care organisations and among other stakeholders.

E. Whilst the Biomedical Research Centres and Units, and now the Academic Health Science Centres, are promising innovations [94] and there is a wider set of funding streams than originally proposed in *Best Research for Best Health*, it is not entirely clear how far there will be sufficient resources to allow research to be undertaken throughout the NHS in a way that is likely to increase its absorptive capacity and hence its eventual use of research. Furthermore, population health literacy programmes are likely to need further development.

F. In the 2008 Research Assessment Exercise, pressure from the DH/NHS research system, and subsequently from the NIHR, helped address many of the previous difficulties that had existed at the interface between the higher education research system (with its norms of assessing research according traditional academic criteria) and researchers working in, and for, the health care system. Furthermore, there is now growing recognition within new NIHR institutions (such as the Biomedical Research Centres and the Collaborations for Leadership in Applied Health Research and Care) of the value of the contributions from different disciplines. Nevertheless, there is probably still a need for mechanisms to ensure that the work of health researchers is assessed ex post on the basis on which it was commissioned and funded rather than according to other criteria.

In summary, despite the considerable progress described above, and in Table 2, it also clear there are still some problems at the interfaces, and a need for resources to develop mechanisms to address them.

### 3. Funding implications and accountability

It is an obvious, but vital, lesson that additional money is very important in being able to undertake additional applied research whilst maintaining the funding for basic science. At one level this is obvious, but the comparison offered here between the Rothschild reforms and the NIHR/Cooksey reforms provides further empirical evidence to support the notion, noted by Cooksey in relation to the reforms in Canada [58], that reforms will go more smoothly when there are additional resources rather than a redistribution. Furthermore, in any system transparency and accountability mechanisms are very important as are appropriate incentives.

In the English health research system as now created following the various reforms, it remains to be seen whether the current arrangements will be able to cope with the likely reduction in UK Government research funding in the future.

## Conclusions

Substantial claims are being made that the latest NIHR strategy has largely become a reality. Our combined stakeholder and historical analysis has enabled us to examine how far the current system has built on previous initiatives, and how far it addresses previously unresolved concerns articulated by the principal stakeholders in the health research system. The analysis indicates that the latest strategy for publicly funded health research in England sensibly both builds on recent progress and tackles acknowledged weaknesses. The strategy goes a considerable way to meeting the needs of medical academics, patients and industry through a system that should improve and simultaneously expand translational, clinical and applied health research, and increase the extent to which research is then used in the health care system. It is doing this in various ways, including building research networks and infrastructural support, increasing efficiency in recruiting research subjects, and developing new funding streams and more institutional capacity for conducting the necessary research.

Analyses of previous reforms demonstrate the complexity of meeting and reconciling the needs of the ever-increasing range of stakeholders. Attempts to get researchers and potential research users to work together face recurring difficulties. The task of co-ordinating the whole enterprise must be given sufficient recognition and resourcing, as must the necessary organisational arrangements and interface mechanisms, which greatly vary with different types of research. Considerable progress is being made in addressing these issues by the new NIHR and also, following the Cooksey Review, by the strategy for the whole health research system in England. But, inevitably, some issues will need further attention.

The new strategy has also been remarkably successful in increasing the funding for health research in England. But, and especially in the context of the budget constraints in the UK public sector from 2010 onwards, it is increasingly important that the health R&D system is seen to be undertaking and producing research that meets the needs of the many stakeholders now involved. This will be necessary to ensure that the health research system retains their continuing commitment. Researchers as core stakeholders in a research system often emphasise the focus on research utilisation should be balanced with an understanding of the creative and often unpredictable nature of research and its impact. The additional funding has thus far been very important in sustaining the support of the academic community who believe that beyond the instrumental value of addressing specific stakeholders' needs, research has inherent value as a valid and reliable way to advance knowledge in human experience.

The progress so far achieved by the reforms to the health research system in England suggests that the needs of a wide range of stakeholders can be met. We therefore conclude that this approach could usefully inform attempts elsewhere to develop health research systems that are similarly responsive.

## Competing interests

The authors declare that they have no competing interests.

## Authors' contributions

SH developed the original idea for the article based on working with Maurice Kogan and Mary Henkel to produce an updated second edition of their evaluation of the first reforms to English health R&D. SH and SK also drew on their experience of contributing to the development of the framework for health research systems produced by the Research Policy and Cooperation department of WHO. NM drew on long experience of commenting on the organisation of health research systems and work with SK on health research impacts and communication. BS drew on experience as a member of the senior staff implementing the reforms of the early 1990s, and subsequently as an analyst of various reforms. All authors contributed to the development of the ideas and to successive drafts of the article. All authors read and approved the final manuscript.
